# Implementation of a National Antimicrobial Stewardship Training Programme for General Practice: A Case Study

**DOI:** 10.3390/antibiotics14020148

**Published:** 2025-02-03

**Authors:** Donna Cooper, Claire Stevens, Conor Jamieson, Ming Xuan Lee, Ruth Riley, Bharat Patel, Jade Meadows, Parmjit Kaur, Obiageli Okolie, Kieran Hand, Donna M. Lecky

**Affiliations:** 1 Black Country Integrated Care Board, Wolverhampton WV1 1SH, UK; 2 Centre for Medicines Optimisation, School of Allied Health Professions and Pharmacy, Keele University, Keele ST5 5BG, UK; c.l.t.stevens@keele.ac.uk (C.S.); bharat.patel4@nhs.net (B.P.); 3 National Health Service England, London SE1 8UG, UK; 4 Primary Care and Interventions Unit, UK Health Security Agency, Gloucester GL1 1DQ, UKobiageli.okolie@ukhsa.gov.uk (O.O.)

**Keywords:** antimicrobial stewardship, TARGET toolkit, antimicrobial resistance, general practice

## Abstract

Background: Approximately 71% of antibiotics in England are prescribed in general practice settings. Whilst there are various impactful training resources available to support clinicians in antimicrobial stewardship (AMS) activities, implementation, reach, and uptake affect how successful they are nationally. This case study explores the feasibility, acceptability, and usefulness of embedding the TARGET (Treat Antibiotics Responsibly, Guidance, Education and Tools) AMS training into a local incentive scheme. Method: Black Country Integrated Care Board (ICB) invited a representative from all associated general practises to a TARGET AMS training event; attendance was linked to a local incentive scheme. Data were collected via a pre- and post-workshop survey capturing TARGET toolkit knowledge, AMS attitudes and behaviours, training feedback, and intention to implement AMS behaviours. Descriptive analyses were conducted. Results: 157 and 101 attendees completed the pre- and post-session surveys, respectively. In total, 89% agreed that attending the session was a good use of their time. The proportions of attendees stating an intention to use the TARGET toolkit and implement a range of AMS strategies following the session were high (TARGET Toolkit: >82%, AMS strategies: >90%). Most attendees planned to implement these actions within 3 months (47%) or within 3–6 months (30%). Conclusion: Results suggest that embedding the training into a local incentive scheme is a viable implementation approach in extending training reach. Although the impact on prescribing rates is not yet available, the high engagement and intention to implement AMS strategies observed should inspire confidence in this approach to training implementation.

## 1. Introduction

Antimicrobial resistance (AMR) remains one of the top priorities for global public health; it is estimated that in 2019, AMR was associated with 4.95 million deaths worldwide [1,2]. The misuse of antimicrobial medicines is a major driver of AMR, which includes underusing, overusing, and inappropriate use [2].

Antimicrobial stewardship (AMS) is defined by the World Health Organisation as “a systematic approach to educate and support health care professionals to follow evidence-based guidelines for prescribing and administering antimicrobials” [3]. A range of AMS interventions have been implemented across England’s primary and secondary care pathways to address AMR [4]. Previous research has demonstrated that the improvement in antibiotic prescribing in English primary care could be facilitated by the national implementation of interventions, such as training in the interactive use of leaflets [5]. In England, the TARGET (Treat Antibiotics Responsibly, Guidance, Education and Tools) toolkit [6] has been designed to support primary care clinicians to implement effective AMS activities. A randomised controlled trial evaluation in routine general practice showed that a one-hour TARGET workshop given by local GPs, microbiologists, or medicine managers, combined with demonstrating the resources, significantly reduced antibiotic use [7]. However, there has been inconsistent implementation of the toolkit across England [8].

The COVID-19 pandemic and the resultant changes to the healthcare landscape have brought additional challenges in tackling AMR. Although community antibiotic use decreased in the earlier stages of the pandemic [9], more recent data suggested a slight increase in primary care prescribing [10], particularity for remote consultations [11] suggesting a need to re-strengthen current AMS interventions.

In October 2022, UKHSA, in collaboration with the National Health Service England (NHSE), initiated a national roll out of TARGET AMS training using a realist approach [12] to implementation. This case study examines the feasibility, acceptability, and usefulness of the approach taken by the NHS Black Country Integrated Care Board within the Midlands region of England.

## 2. Results

Participation was high; 82% (149/181) of general practice representatives attended the event. A total of 157 attendees (12 advanced nurse practitioners, 94 GPs, 49 pharmacists, and 2 other) completed the pre-session survey, and 101 completed the post-session survey (10 advanced nurse practitioners, 57 GPs, 34 pharmacists) with responses from representatives of all 33 PCNs and 181 general practises in the ICB.

### 2.1. Acceptability and Usefulness of the AMS Training Event

In total, 89% (90/101) agreed that attending the training session was a good use of their time; 88% (89/101) were well engaged throughout the training, with 88% (89/101) saying it was easy for them to become actively involved. Attendees understood (99%, 100/101) and related to (96%, 97/101) the learning objectives.

Prior to the session, two-thirds (66%, 104/157) of attendees who completed the pre-session survey were already aware of the TARGET toolkit; over half (55%, 57/104) indicated that the toolkit is more useful than other available AMS resources. Following the session, attendees were aware of where to find the TARGET toolkit (98%, 99/101) and how to use it (96%, 97/101).

### 2.2. Attitudes and Knowledge Towards AMR

Attitudes towards, and understanding of, AMR did not greatly change between the pre- and post-session surveys. Over 95% positive responses were observed to questions about awareness of AMR and prescribers’ role in contributing to and addressing it.

**Figure 1:**
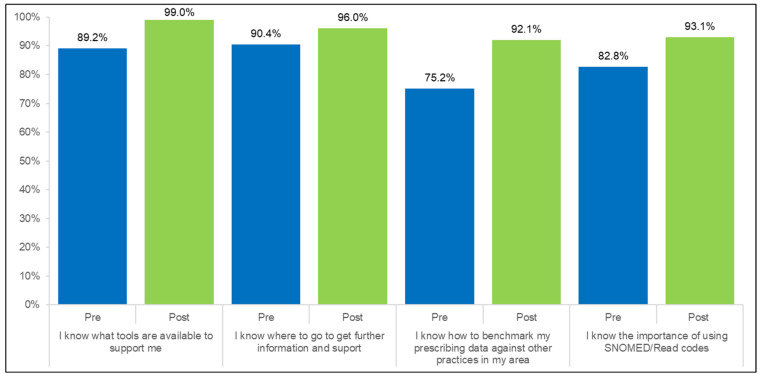
Participant pre- and post-training knowledge and attitudes towards AMS tools and support (Pre: *n* = 157; Post: *n* = 101).

A 16.9 percentage point (pp) increase was observed in attendees knowing how to benchmark prescribing data (92%, 93/101); a 10.3 pp increase was observed in attendees understanding the importance of using clinical codes (93%, 94/101) (Figure 1).

### 2.3. Use of AMS Tools and Strategies

**Figure 2:**
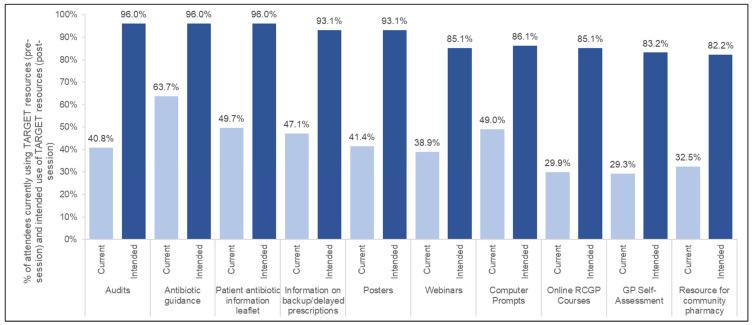
Current and intended use of Target toolkit resources (current: *n* = 157; intended: *n* = 101).

Following the training session, a large increase was observed in attendees’ intentions to implement a range of AMS tools/resources within their practice (Figure 2); AMS tools with the highest intention to use included audits (40.8% before vs. 96%, after), patient information leaflets (49.7% before vs. 96% after), and antibiotic guidance (63.7% before vs. 96% after).

**Figure 3:**
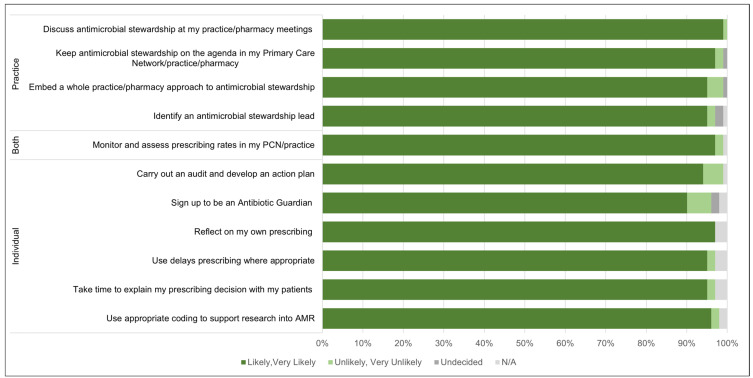
Intended use of AMS strategies (*n* = 101).

A high intention to embed wider AMS strategies (Figure 3) was also observed post-training; 99% (100/101) of attendees plan to discuss AMS at practice meetings; 95% (96/101) intend to take a whole practice approach to AMS.

### 2.4. Practice Implementation of an Antimicrobial Stewardship Action Plan

**Figure 4:**
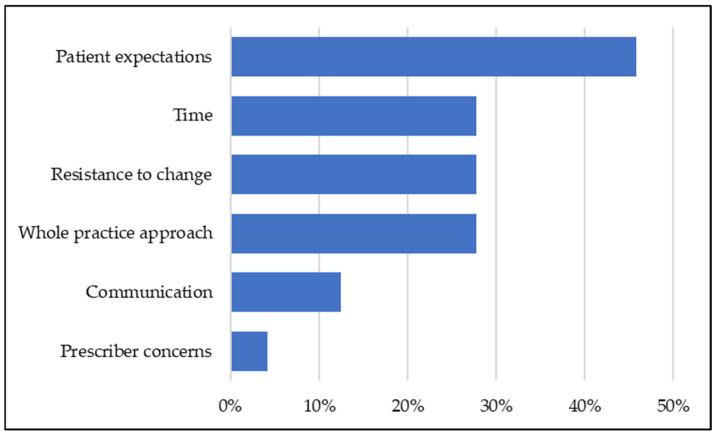
Anticipated barriers to implementing learning (*n* = 72).

In total, 97% (98/101) of attendees agreed that they will be able to apply actions from the training session in their practice; 96% (97/101) anticipate they will see positive results from these actions. The majority of attendees plan to implement actions within 0–3 months after the event (47%, 47/101) or within 3–6 months (30%, 30/101). A total of 72 attendees responded to a free text question in relation to the anticipated barriers to implementing learning, following training. Responses were coded (Figure 4) with the highest perceived barrier being managing patient expectations about receiving antibiotics (46%, 33/72). Patient expectations ranged from historical prescribing for the same, or similar symptoms, to inconsistent prescribing between prescribers within and outside the practice. Prescriber concerns was the least reported perceived barrier (4%, 3/72).

## 3. Methods

### 3.1. The Intervention: TARGET AMS Training

The TARGET AMS training workshops utilise the COM-B model [13] approach, providing clinicians with the capability, opportunity, and motivation to facilitate appropriate prescribing. The training demonstrates the link between antibiotic prescribing and resistance, and how reducing antibiotic prescribing can reduce antibiotic resistance, patients’ future expectations for an antibiotic, and re-consultation rates. Workshops utilise pre-prepared presentation materials and are delivered by a trained facilitator. A breakdown of the TARGET training and all training materials can be found on the ‘TARGET tools to train prescribers’ section of the TARGET website [6].

#### Suggested National Training Approach

**Figure 5:**
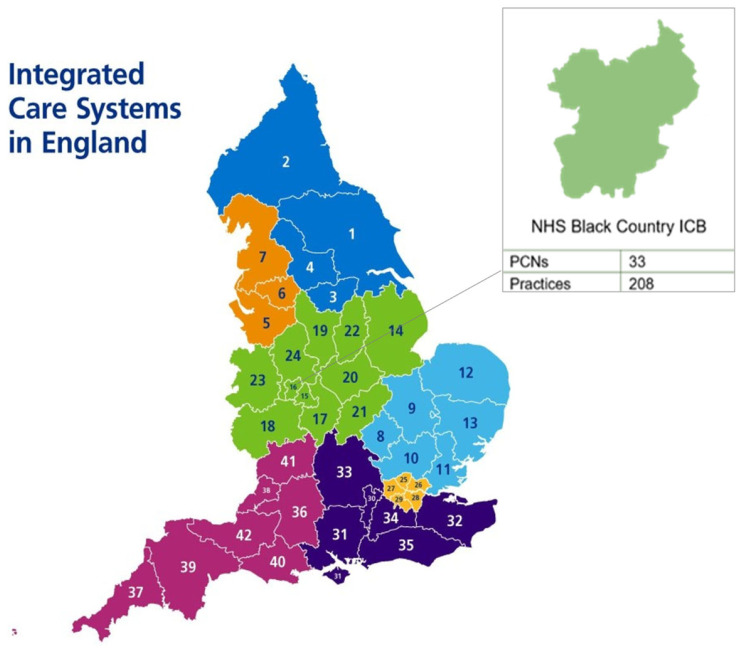
Location for TARGET training case study—NHS Black Country ICB. Source: https://www.england.nhs.uk/integratedcare/integrated-care-in-your-area/ (accessed on 24 September 2024).

England is divided into 7 National Health Service (NHS) England regions, each hosting between 3 and 11 Integrated Care Boards (ICBs) responsible for planning health services for their local population, working with local providers of NHS services, such as hospitals and primary care general practice (organised into primary care networks [PCNs]) and local councils (Figure 5—regional map) [14]. Each NHS England region has both an AMS lead and an Infection Prevention Control (IPC) lead.

### 3.2. The Black Country ICB

The Black Country ICB is situated in the Midlands region of England (Figure 1). The Midlands is the largest English region comprising 11 ICBs and a population of approximately 10.8 million people (ONS mid-year population estimate) [15]. Antibiotic prescribing rates in the Midlands follow national and seasonal trends, overall. ePACT2 is a prescription data dashboard that allows authorised users to look at high-level data summaries down to individual prescription item and patient-level detail [16]. Data obtained from ePACT2 on antibiotic prescriptions in primary care show that the Midlands has the 4th highest prescribing rate across the seven regions at 53 items per 1000 individuals in April 2024. Rates in the Midlands are closer to the highest prescribers (54 items per 1000 individuals) rather than the lowest prescribers (41 items per 1000 individuals) [10]. The Black Country ICB was formed in 2022 from the merging of four former clinical commissioning groups: Dudley, Sandwell, Walsall, and Wolverhampton, now referred to as ‘Places’. The care system population is highly diverse with higher proportions of Black, Asian, and Minority Ethnic communities than the national average of 14% in Sandwell (40%), Walsall (28%), and Wolverhampton (39%). The ICB has the second most deprived integrated care system population in England with 28 to 60% of the population living in the most deprived areas [17].

**Figure 6:**
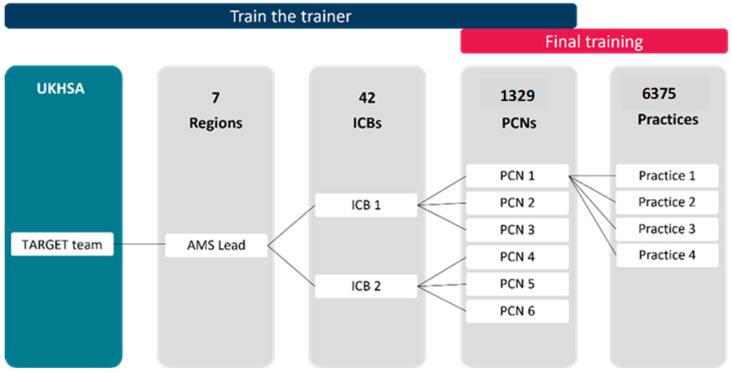
Proposed training cascade model.

Although a cascade model of implementation (‘Train the trainer’), coordinated by NHSE AMS regional leads, was proposed (Figure 6), the realist approach allowed for flexibility, acknowledging organisational and geographical differences within each region. For logistical reasons, ICBs were approached individually to consider training implementation.

### 3.3. The NHS Black Country ICB Implementation Plan

The NHS Black Country ICB took the approach of embedding the TARGET AMS training into a localised incentive scheme, 25% of which focused on AMS. To qualify for a 25% incentive payment for the AMS element of the scheme, practises were required to meet the following criteria (Table 1):

The face-to-face AMS training event, organised by NHS Black Country ICB in collaboration with Keele University, and supported by the UK Health Security Agency (UKHSA) TARGET team and the NHSE regional AMS lead, took place on Wednesday 14 June 2023; at least one representative from each of 181 ICB general practises was invited to attend. Training was delivered by a practice clinical pharmacist (BP) and the Midlands AMS regional lead (CJ). The agenda for the day is available in Supplementary File S1.

Two key priorities of the face-to-face event were to (1) take primary healthcare professionals out of their ICB ‘Place mindset’ and encourage greater cross-ICB working, thus promoting the sharing of AMS knowledge and best practice, and (2) to capitalise on the interactive elements of the clinical scenarios and action planning in the TARGET training.

The TARGET acute cough and acute sore throat clinical scenarios were used. In addition to the TARGET training learning objectives, key messages included ‘shorter is better’—a reference to minimising the duration of antibiotic prescriptions [19,20]; consistency of approach across all prescribers in the practice; importance of accuracy in coding for diagnosis; use of safety netting and delayed prescriptions; and use of the FeverPAIN [21] score to assess sore throat. In addition, a series of ‘how-to’ videos were created for the event and shared with attendees; these included the following:

1. Using the FeverPAIN clinical score and copying information into the patient record.
2. Using AccuRx to send TARGET patient information leaflets electronically.
3. Printing TARGET patient information leaflets via System One.
4. Printing TARGET patient information leaflets via EMIS.

In total, 25 facilitators supported action planning, encouraging attendees to have AMS as a standing agenda item in practice meetings, and to take a whole practice approach to the planning and implementation of AMS strategies, including the use of the TARGET toolkit. Attendees were provided with detailed practice-level data on key prescribing metrics, compared to ICB and national benchmarks to facilitate the identification of priority areas for practice-level action planning.

### 3.4. Data Collection and Analysis

Descriptive analyses were conducted on data collected via pre- and post-workshop surveys. The pre-workshop survey captured attendee details, knowledge and use of the TARGET toolkit, and AMS attitudes and behaviours; the post-workshop survey captured training feedback, intention to implement actions, and AMS attitudes and knowledge.

## 4. R4. Discussion

### 4.1. Summary

We found that embedding the TARGET AMS training into a local incentive scheme was a successful means of delivering the training to a large number of general practises in an area. Attendees left the event feeling more knowledgeable about how to access local and national prescribing data, had increased intentions to use a range of AMS tools within their practice, and were highly motivated to implement an AMS plan.

### 4.2. Comparison to the Literature

Successful intervention implementation is complex, with various implementation and behavioural frameworks available to guide the process. One such behavioural framework, the COM-B [13], states that an individual must have the capability and opportunity, as well as the motivation, to engage in said behaviour. Alongside this, implementation scientists suggest that ensuring intervention fit between a service and the intended healthcare setting is critical for implementation success [22].

The training day provided attendees with the opportunity to improve knowledge around the issue of AMR, whilst the training itself facilitated clinical capability to embed AMS activities in practice. The provision of localised prescribing data prior to the event enabled training to be delivered within the wider ICB context, supporting implementation fit. Peer delivery is well recognised and regarded [23]; therefore, training delivery by the regional AMS lead and a local practice clinical pharmacist who already uses and promotes the TARGET toolkit not only made the translation of theory into practice conceivable but may also have fostered a greater sense of ownership among attendees. GPs and nursing staff have also delivered training in other regions. The selection of topics for case studies (sore throat, acute cough) was designed to support current clinical priorities.

Local incentive schemes have shown to be effective in addressing relevant local needs and cost saving to the NHS [24]. Embedding TARGET training and action planning into the AMS element of a local incentive scheme, as in our case study, may have resulted in increased attendance. Cochrane review findings indicate that financial incentives may be effective in changing healthcare professional behaviours and, in turn, patient outcomes [25].

### 4.3. Strengths and Limitations of the Approach

A collaborative approach to implementation proved extremely successful. Data show that cross-organisational/government implementation has the potential to maximise the impact of an intervention by providing opportunities to address challenging issues, ensuring a long-term and preventative approach, to share good practice and expertise as well as potential cost savings [26].

AMS training is not a mandatory requirement for clinicians in the UK, though the TARGET training fills a much needed gap in knowledge. Embedding the training as part of a local incentive scheme helps ensure wider training reach and uptake, and consistent messaging across the ICB.

Prior to the event, there was a preconceived risk that having 150+ attendees could have an adverse effect on engagement, participation, and opportunity to put training into a local context. However, post-event survey results indicate that this risk was not realised, with verbal feedback indicating that this was largely due to how the training was tailored and by having opportunities to share knowledge and to interact with peers, something that is of importance to GPs [27]. It was notable at the event that a deliberate lack of a fixed seating arrangement by Place or PCN fostered a spontaneous mix of delegates from different ‘Places’ at each table able to share best practice from a Place perspective. Attendance at the event also reflected the evolving skill mix of prescribers within primary care teams with 40% of non-medical prescribers attending (advanced nurse practitioners [7.6%], pharmacists [31%], and ‘other’ [1.4%]. Evidence suggests a high level of patient trust towards their healthcare professionals (HCPs) [28] and similar levels of patient expectations on GPs and non-medical prescribers (NMPs) [29,30]; therefore, taking a whole practice approach to AMS learning is crucial for successful outcomes. NMPs also report less time constraints and a strong emphasis on patient education and alternatives to antibiotics [29].

In addition to the promising intentions indicated in the post-session survey, there are several additional benefits to taking an ICB-wide approach to the cascade of training.

1. Fidelity of the training is not lost through a cascade, providing all practises with consistent messages and information.
2. Shared learning experience provides prescribers the opportunity to share knowledge and best practice outside of their day-to-day setting.
3. General practises within the ICB are at the same stage of action planning and implementation, making it easier for Medicines Optimisation teams to provide support and follow up on progress.
4. The ICB can promote collective AMS actions, encouraging a collaborative approach.
5. Reduction in individual administrative burden and time in comparison to the train-the-trainer approach.

Some considerations that ICBs should give before taking this approach include the following.

1. Budget to hire an appropriate venue for the intended audience.
2. Availability of an appropriate venue in the area (size, location, transport links/parking, cost, dates).
3. Availability of attendees, particularly when one date is offered. This could leave sole practitioners at a disadvantage if unable to attend.
4. Short-term administrative burden and support for arranging a large-scale event.
5. Development of a pre- and post-event action plan to maximise the impact.
6. Presenter knowledge, experience, and confidence.

Intended actions following the event are encouraging, and there is a strong belief from attendees that they will see a positive result from implementing action plans. Although barriers to implementation strategies were identified, these were detailed with associate mitigation strategies, suggesting attendees felt capable of addressing potential hurdles. The majority (95%) of attendees intend to implement a whole practice approach; however, resistance to change, particularly from peers, was highlighted by prescribers when describing anticipated barriers. Research suggests that audit feedback, comparison with peers, discussion, and action planning are essential for onward behaviour change [27,31,32,33], and it is highly recommended that attendees form an action plan in collaboration with other practice members following the session to increase buy-in and capitalise on opportunities to optimise antibiotic prescribing.

## 5. Conclusions

The comprehensive implementation of decision support toolkits, such as TARGET, is needed to ensure support for the ongoing battle with AMR. The collaborative working model has been successful in extending training reach, and embedding AMS training into local incentive schemes may help with staff engagement.

Although it is too early to measure the impact on prescribing rates, high engagement, and intention to implement AMS strategies and use TARGET resources indicate that an ICB-wide training event is a feasible and acceptable approach to cascading training, with attendees leaving motivated and capable of developing and implementing action plans.

Post-survey findings suggest that attendees left the training day highly motivated to implement AMS strategies; however, more work needs to be performed to see what strategies were implemented and if they led to a change in prescribing.

## Figures and Tables

**Table 1:** The NHS Black Country ICB qualifying criteria for incentive payments.

Criteria	Proportion of Payment Total
Practises must identify a prescribing (medical or non-medical) AMS lead who must be willing to be responsible for driving change within the practice.	2%
The AMS lead must attend the face-to-face event (as described below)	5%
All practises to submit an action plan detailing how they will implement TARGET toolkit resources within the practice—deadline for submission to the ICB Medicines Optimisation/Prescribing Support Team is 31 July 2023.	3%
Those practises not meeting the NHS England target for total volume of antibiotics, 0.871 items/STAR-PU [18], to either meet the target or make a minimum of a 10% reduction from baseline April 2022 to March 2023 (payment based on sliding scale achievement).	15%

## Data Availability

The original contributions presented in this study are included in this article. Further inquiries can be directed to the corresponding author.

## Supplementary Materials

The following supporting information can be downloaded at: https://www.mdpi.com/article/10.3390/antibiotics14020148/s1, File S1: Agenda for the Antimicrobial Stewardship Workshop.
