# Supplementary material for: Implementation of a National Antimicrobial Stewardship Training Programme for General Practice: A Case Study
Source: Antibiotics (Basel). 2025 Feb 3;14(2):148. doi: 10.3390/antibiotics14020148 (PMC11851554; doi:10.3390/antibiotics14020148)
Supplement: Supplementary file 1 [file antibiotics-14-00148-s001.zip › antibiotics-3306787-supplementary.pdf]

NHS
Black Country
Integrated Care Board

TARGET
Keep Antibiotics Working

Keele University
Centre for Medicines Optimisation

Antimicrobial Stewardship Workshop

A half-day workshop focusing on the use of the TARGET antibiotics toolkit to assist practices to promote good antimicrobial stewardship (AMS) and support participation in the AMS element of the incentive scheme.

Wednesday 14th June at Bescot Stadium, Bescot Crescent, Walsall WS1 4SA, 12 to 4.30pm

Certificate of attendance available to use for CPD.

Please note, there is an interactive element to the workshop that requires a charged laptop with secure VPN access to clinical systems

Please complete the TARGET Self Assessment Checklist prior to attending the workshop.

Speakers

- ***Bharat Patel is a Practice-based Clinical Pharmacist*** in Walsall, clinical mentor for the NHSE GP Pharmacists Training Pathway and current Chair of the Midlands Pharmacy Practice Network. Bharat was previously Head of Medicines Optimisation for Walsall CCG
- ***Dr Conor Jamieson is the Regional Antimicrobial Stewardship Lead*** for NHS England & Improvement for the Midlands region. He has previously worked as an antibiotic pharmacist in hospitals in the West Midlands since 2002. He is the current chair of the BSAC Drug Stability Testing Programme, a part of the BSAC OPAT Initiative.
- ***Catherine Hayes*** is the TARGET Project Manager
- ***Ruth Riley*** is TARGET Training roll out Programme Manager

AGENDA

Time	Sessions and Activities	Speaker
11.45 onwards	Arrival and registration	
12.00 to 1.00 pm	Lunch and networking	Representatives attending from CPCS, enhanced services & Pharmacy First
1.00 to 1.05 pm	Introduction to the Session	Bharat Patel
1.05 to 1.15 pm	Accessing the Data Hub	Annabel Smith, BC ICB Data Team
1.15 to 1.35 pm	Tour of the TARGET website and talk of latest updates	Ruth Riley and Catherine Hayes, UKHSA
1.35 to 3.00 pm	Application of TARGET resources in specific therapeutic areas, with a focus on acute cough & sore throat	Dr Conor Jamieson
3.00 to 3.15 pm	Break for Tea, Coffee & Biscuits	
3.15 to 4.00 pm	Workshop: Creating the Action Plan and feedback session	Bharat Patel (facilitated by Ruth Riley, Conor Jamieson, the Medicines Optimisation Team and Prescribing Support Team)
4.00 to 4.30 pm	Sharing the learning: introduction to the outreach pack and key learning points	Bharat Patel<br>Feedback and evaluation

Supplementary File S1. Agenda for the Antimicrobial Stewardship Workshop.
